# Silencing of *Nicotiana benthamiana Neuroblastoma-Amplified Gene* causes ER stress and cell death

**DOI:** 10.1186/1471-2229-13-69

**Published:** 2013-04-27

**Authors:** Jae-Yong Lee, Sujon Sarowar, Hee Seung Kim, Hyeran Kim, Inhwan Hwang, Young Jin Kim, Hyun-Sook Pai

**Affiliations:** 1 Department of Systems Biology, Yonsei University, Seoul 120-749, Korea; 2 Division of Molecular and Life Sciences, Pohang University of Science and Technology, Pohang 790-784, Korea; 3 School of Life Sciences and Biotechnology, Korea University, Seoul 136-701, Korea

**Keywords:** bZIP28, ER stress gene expression, Promoter-GUS fusion, Protein transport assay, Virus-induced gene silencing

## Abstract

**Background:**

*Neuroblastoma Amplified Gene* (*NAG*) was identified as a gene co-amplified with the N-*myc* gene, whose genomic amplification correlates with poor prognosis of neuroblastoma. Later it was found that NAG is localized in endoplasmic reticulum (ER) and is a component of the syntaxin 18 complex that is involved in Golgi-to-ER retrograde transport in human cells. Homologous sequences of *NAG* are found in plant databases, but its function in plant cells remains unknown.

**Results:**

*Nicotiana benthamania Neuroblastoma-Amplified Gene* (*NbNAG*) encodes a protein of 2,409 amino acids that contains the secretory pathway Sec39 domain and is mainly localized in the ER. Silencing of *NbNAG* by virus-induced gene silencing resulted in growth arrest and acute plant death with morphological markers of programmed cell death (PCD), which include chromatin fragmentation and modification of mitochondrial membrane potential. NbNAG deficiency caused induction of ER stress genes, disruption of the ER network, and relocation of bZIP28 transcription factor from the ER membrane to the nucleus, similar to the phenotypes of tunicamycin-induced ER stress in a plant cell. *NbNAG* silencing caused defects in intracellular transport of diverse cargo proteins, suggesting that a blocked secretion pathway by NbNAG deficiency causes ER stress and programmed cell death.

**Conclusions:**

These results suggest that NAG, a conserved protein from yeast to mammals, plays an essential role in plant growth and development by modulating protein transport pathway, ER stress response and PCD.

## Background

Programmed cell death (PCD) is a genetically defined process associated with distinctive morphological and biochemical characteristics, and is an integral part of the life cycle of multicellular organisms [1,2]. In plants, PCD occurs during developmental processes and in response to abiotic and biotic stresses [3,4]. The major PCD signaling pathways involve mitochondria and plasma membrane receptors, although it has recently been shown that ER stress caused by impaired ER function can also induce apoptotic pathways in animals and plants [5-7].

The ER performs several important functions, including protein targeting and secretion, vesicle trafficking, and membrane biogenesis, and its proper function is essential to cell survival. Perturbations in ER homeostasis disrupt folding of proteins, leading to accumulation of unfolded proteins and protein aggregates. This condition is called ER stress and is detrimental to cell survival [5,8]. Under conditions of ER stress, a cell activates a signal transduction pathway termed the unfolded protein response (UPR) to limit the damage and maintain ER homeostasis [7,9,10]. Prolonged and excessive ER stress induces apoptosis, evidenced by DNA fragmentation, cytochrome *c* release, and induction of caspase activity [11]. In mammals, the ER stress-induced apoptotic pathway involves cross-talk between ER and mitochondria through Bcl-2 family members Bcl-2, Bax and Bak, which are localized in both mitochondria and ER [5]. Recent reports suggest that in animal cells, the mitochondrial apoptotic pathway mediated by Apaf-1 is an integral part of ER stress-induced apoptosis [2,5,11]. In plants, treatment with tunicamycin, an inhibitor of N-linked protein glycosylation, and cyclopiazonic acid, a blocker of plant ER-type IIA calcium pumps, elicits ER stress, followed by activation of PCD with typical apoptotic morphology [6,12]. However, the mechanisms of UPR and ER stress-induced PCD in plants remain largely unknown. One mechanism of ER stress response in *Arabidopsis* involves membrane-associated bZIP transcription factors; following ER stress, bZIP28 and bZIP60 are processed and their released N-terminal domains are translocated to the nucleus to upregulate the expression of ER stress response genes including BiPs (ER chaperones), BI-1 (Bax-Inhibitor 1), PDI (protein disulfide isomerase), and calnexin [13-15].

Genomic amplification (3 to 300 copies) of N-*myc* oncogene in human neuroblastoma correlates with aggressive tumor growth and poor prognosis [16]. *Neuroblastoma Amplified Gene* (*NAG*) was first identified as a gene co-amplified with the N-*myc* gene, although *NAG* is widely expressed in normal human tissues and its homologous sequences are found in plant databases [16,17]. Recently, it has been shown that NAG is localized in ER and is a component of the syntaxin 18 complex that is involved in Golgi-to-ER retrograde transport using human cell lines [18]. In this study, we investigated *in planta* functions of NAG in *Nicotiana benthamiana*. We showed that *N. benthamiana* NAG (NbNAG) played a role in protein transport pathway, and NbNAG deficiency caused ER stress and cell death, suggesting its essential role in plant growth and survival.

## Results

### Identification of NbNAG

Functional genomics using virus-induced gene silencing (VIGS) revealed that silencing of the *N. benthamiana* homolog of *Neuroblastoma Amplified Gene* (*NAG*), designated *NbNAG*, results in growth arrest and acute plant death. The ~7.4 kb full-length *NbNAG* cDNA encodes a polypeptide of 2,409 amino acids with a predicted molecular mass of 270,589.33 Da (Additional file 1: Figure S1). Database searches identified closely related genes in human, mouse, zebrafish, *C. elegans*, *Arabidopsis* and rice. The *NAG* homolog encodes a protein of ~2,400 amino acids in *Arabidopsis* (At5g24350), rice (NP_001066451), and human (NP_056993), and is a single-copy gene in all three genomes. Our analyses showed that there is no null mutant of *NAG* in *Arabidopsis* T-DNA insertion lines. The NbNAG protein contains the Sec39 domain (residues 595*–*1126) that has been implicated in ER-Golgi trafficking in yeast [19]. Overall, NbNAG shows 49%, 42% and 29% sequence identity to the NAG homologs from *Arabidopsis*, rice, and human, respectively. NbNAG sequence was aligned with those of the NAG homologs from *Arabidopsis* and rice (Additional file 1: Figure S1).

### ER localization of NbNAG in tobacco BY-2 cells

**Figure 1:**
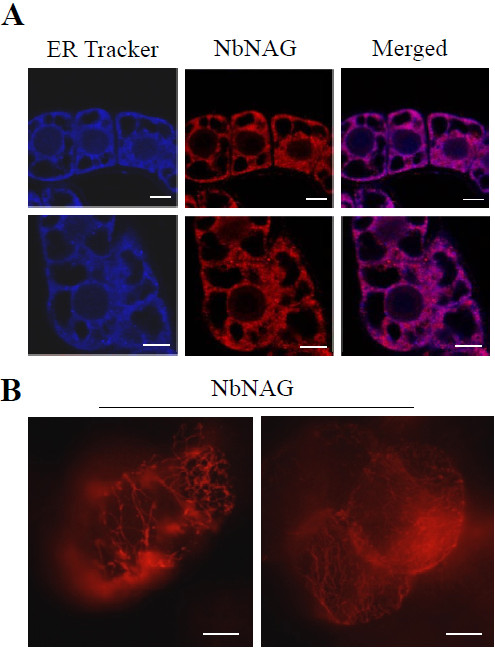
**ER localization of NbNAG in tobacco BY-2 cells.** A. BY-2 cells were immunolabeled with anti-NbNAG antibodies and briefly stained with ER. Tracker™ Blue-White DPX as an ER marker for observation with confocal laser scanning microscopy. Scale bars: 10 μm. B. After immunolabeling, the BY-2 cells were examined by fluorescence microscopy. Fluorescent and bright field images are shown. Scale bars: 10 μm.

To determine subcellular localization of NbNAG, we performed immunolabeling experiments in tobacco BY-2 cells using anti-NbNAG antibodies (Figure 1). After immunolabeling with anti-NbNAG antibodies, BY-2 cells were briefly stained with ER Tracker™ Blue-White DPX as an ER marker. Confocal laser scanning microscopy revealed that red fluorescent signals of NbNAG overlapped with blue fluorescence of the ER Tracker near the nuclei and along the network in a typical ER localization pattern in BY-2 cells [20] (Figure 1A). When the BY-2 cells were examined by fluorescence microscopy, they exhibited a network pattern of red fluorescence in the cytosol and in the nuclear periphery (Figure 1B). These results suggest that NbNAG was mainly localized in the ER, consistent with the ER localization of the yeast and mammalian NAG [18,19].

### Expression of *Arabidopsis NAG*

**Figure 2:**
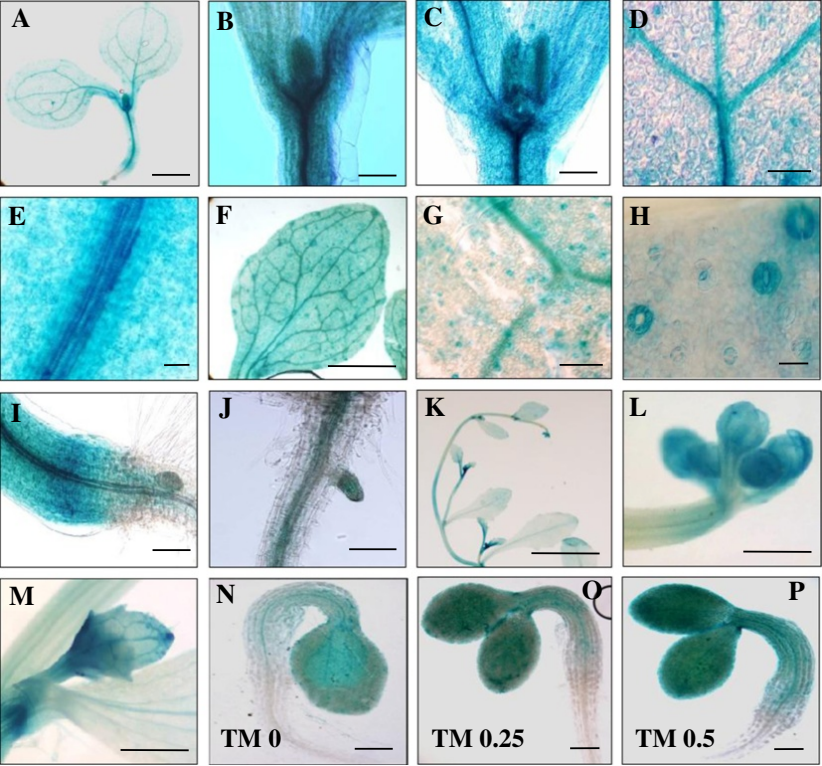
**Histochemical localization of *AtNAG *promoter-*GUS *expression in *Arabidopsis*.** In *Arabidopsis* transgenic plants carrying the *Arabidopsis NAG* (*AtNAG*) promoter**-***GUS* fusion gene, GUS staining is shown in the following tissues: shoot of a seedling at 4 days after germination (DAG) (**A**); shoot apex and leaf primordia at 4 DAG (**B**) and 7 DAG (**C**); vascular bundles in the cotyledon (**D**) and the hypocotyl (**E**) at 4 DAG; vasculature (**F** and **G**) and stomata (**F** and **H**) of a true leaf at 7 DAG; hypocotyl-root junction at 4 DAG (**I**); root vasculature and emerging lateral root (**J**); flower buds and axillary buds in the reproductive stage (**K**), and enlarged pictures of the flower buds (**L**) and the axillary buds (**M**); a seedling at 2 DAG upon tunicamycin (TM) treatment at 0 (**N**), 0.25 (**O**), and 0.5 μg/ml (**P**). Scale bars: **A** = 2 mm, **B**, **C**, **I**, and **L** = 0.5 mm, **D**, **G**, and **N-P** = 200 μm, **E** and **J** = 100 μm, **F** and **M** = 1 mm, **H** = 20 μm, **K** = 1 cm.

We transformed *Arabidopsis* with a fusion construct between the *Arabidopsis NAG* promoter (1,100 bp) and *GUS* (*AtNAG*p*::GUS*). The 1,100 bp promoter was the maximum size due to a closely located adjacent gene in *Arabidopsis* genome. GUS staining of seedlings demonstrated that *AtNAG* promoter activity was mainly detected in the aerial parts, particularly in shoot apical meristem, leaf primordia, developing vasculature, and stomata (Figure 2A-I). In roots, GUS activity was localized in emerging lateral roots and root vasculature, albeit weakly (Figure 2J). In the reproductive stage, flower buds and axillary buds showed GUS staining (Figure 2K-M). In general, *NAG* expression was concentrated in young aerial tissues containing dividing cells. Interestingly, *AtNAG*p*::GUS* activity in a seedling increased in response to increasing concentrations of tunicamycin (TM), an inhibitor of N-linked glycosylation (Figure 2N-P).

### VIGS phenotypes and suppression of endogenous *NbNAG* transcripts

**Figure 3:**
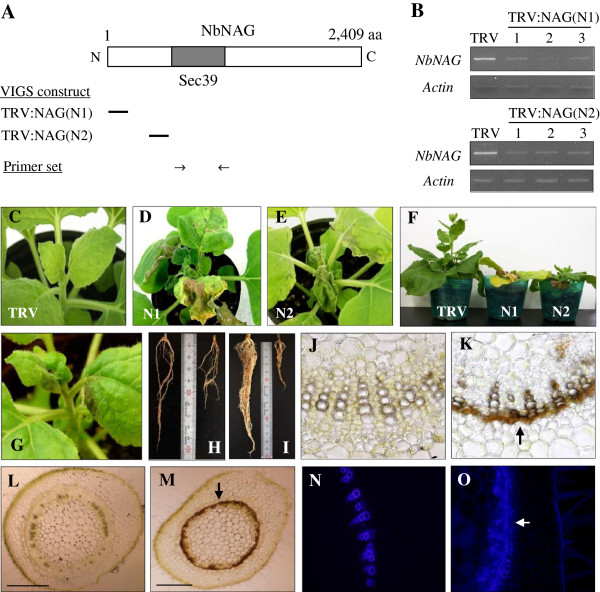
**VIGS constructs, phenotypes, and suppression of the *NbNAG *transcripts. A** Schematic drawing showing NbNAG structure and two VIGS constructs, TRV:NAG(N1) and TRV:NAG(N2), each containing a different *NbNAG* cDNA fragment (indicated by *bars*). The primer set used for RT-PCR is also indicated. **B** Semiquantitative RT-PCR analysis of *NbNAG* transcript levels. Three independent VIGS plants were analyzed for TRV:NAG(N1) and TRV:NAG(N2) lines. The actin mRNA level was included as a control. **C-F** Cell death phenotypes of TRV:NAG VIGS plants. Photographs of the plants were taken at 25 days after infiltration (DAI). **G** Cell death phenotypes of TRV:NAG VIGS plants at 15 DAI. **H** and **I** Root growth of TRV (*left*) and TRV:NAG lines (*right*) at 15 (**H**) and 25 DAI (**I**). **J-M** Hand-cut sections of the petiole of the fourth leaf above the infiltrated leaf (**J** and **K**) and the stem where the fourth leaf above the infiltrated leaf was attached (**L** and **M**) from TRV (**J** and **L**) and TRV:NAG lines (**K** and **M**) at 15 DAI. Brown-colored dead cells in the vasculature are indicated by the *arrows*. Scale bars: 200 μm. **N** and **O** The stem sections shown in (**L** and **M**) were observed under fluorescence microscopy to detect autofluorescent secondary metabolites in TRV (**N**) and TRV:NAG lines (**O**). The *arrow* indicates the autofluorescence around the stem vasculature that undergoes cell death.

For VIGS, we cloned two different fragments of *NbNAG* cDNA into the TRV (Tobacco Rattle Virus)-based VIGS vector pTV00, and infiltrated *N. benthamiana* plants with *Agrobacterium* containing each plasmid. TRV:NAG(N1) and TRV:NAG(N2) contain the non-overlapping 550 bp and 540 bp N-terminal regions of the cDNA, respectively (Figure 3A). The effect of gene silencing on the level of *NbNAG* mRNA was examined using semiquantitative RT-PCR (Figure 3B). Reduced amounts of PCR product were produced in three independent plants from the TRV:NAG(N1) and TRV:NAG(N2) lines compared with TRV control, indicating that the endogenous level of the *NbNAG* transcripts was reduced in those plants. The transcript levels of actin, which served as the control, remained constant.

VIGS with TRV:NAG(N1) and TRV:NAG(N2) constructs resulted in growth arrest and acute plant death (Figure 3C-F). Necrotic lesions were evident in young leaves around the shoot apex at 15–16 days after infiltration (DAI) (Figure 3G). At 25 DAI, the shoot apex was completely abolished with no stem growth or new leaf formation (Figure 3D, E). The lesions progressively expanded, leading to premature death of the plants. Root growth was also affected by *NbNAG* VIGS at 15 and 25 DAI (Figure 3H, I). The petiole of the fourth leaf above the infiltrated leaf, and the stem where the fourth leaf above the infiltrated leaf was attached were cross-sectioned freehand and observed under light microscopy (Figure 3J-M). The localized brown pigment in the petiole (Figure 3K, cf. control in J) and the stem (Figure 3M, cf. control in L) at 20 DAI indicates cell death in the vasculature of TRV:NAG lines, particularly in the cambium. Fluorescence microscopy revealed that TRV:NAG lines accumulated large amounts of autofluorescent secondary metabolites, which were also observed at infection sites during hypersensitive cell death [21], in the stem vasculature (Figure 3O), while TRV control exhibited the fluorescence only in the xylem tracheary elements (Figure 3N).

### Analysis of programmed cell death phenotypes

**Figure 4:**
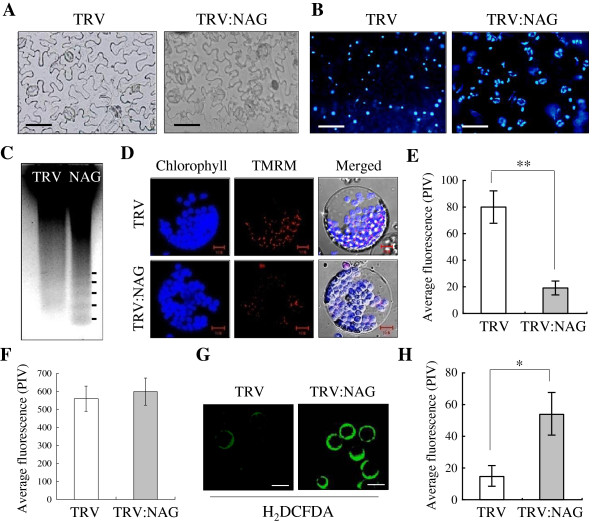
**Phenotypes of programmed cell death. A** Representative light micrographs of the abaxial leaf epidermis of TRV control and TRV:NAG lines (20 DAI). Scale bars: 100 μm. **B** Nuclear degradation. Fluorescence micrographs of abaxial leaf epidermal cells from VIGS lines (20 DAI) after nuclear staining with 4',6-diamidino-2-phenylindole (DAPI; 100 μg/ml). **C** Oligonucleosomal DNA fragmentation. Genomic Southern blotting was performed using total genomic DNA of *N. benthamiana* as a probe. **D-F** Mitochondrial membrane integrity. Leaf protoplasts from VIGS lines (20 DAI) were observed after staining with TMRM (200 nM) (**D**). TMRM fluorescence (**E**) and chlorophyll autofluorescence (**F**) were quantified. Data points represent means ± SD of 20 individual protoplasts. Significant differences between control and other samples were indicated by one (*P*≤0.05) or two (*P*≤0.01) *asterisks*. PIV, pixel intensity values. **G** and **H** ROS production. Leaf protoplasts were incubated with a ROS indicator H_2_DCFDA (2 μM) (**G**). Fluorescence of protoplasts from the VIGS lines was quantified by pixel intensity (**H**). Data points represent means ± SD of 30 individual protoplasts. Scale bars: 50μm.

Although morphology and sizes of abaxial epidermal cells of the TRV:NAG leaves remained normal compared with TRV control (Figure 4A), extension and fragmentation of nuclear chromatin was evident in TRV:NAG lines at 20 DAI (Figure 4B), but not at 10 DAI when lesions were not formed yet (Additional file 1: Figure S2A). DNA laddering was also observed in genomic DNA isolated from the TRV:NAG leaves (Figure 4C). Since nuclear fragmentation and DNA laddering are hallmark features of PCD, these results suggest that silencing of *NbNAG* activates PCD in plants.

During apoptosis in animal cells, activation of the cell death pathway is initiated by modification of mitochondrial membrane permeability [3,4]. Mitochondrial membrane potential of leaf protoplasts from VIGS lines was monitored by TMRM (Tetramethylrhodamine methyl ester) fluorescent probes that accumulate in mitochondria in proportion to the mitochondrial membrane potential [22]. The average TMRM fluorescence of TRV:NAG protoplasts was ~4-fold lower than that of TRV control at 20 DAI, indicating reduced mitochondrial membrane potential (Figures 4D, E, Additional file 1: Figure S5A), but there was no difference in TMRM fluorescence between TRV and TRV:NAG lines at 10 DAI (Additional file 1: Figure S2B, C). Chlorophyll autofluorescence was not affected in TRV:NAG protoplasts at either 10 DAI (Additional file 1: Figure S2B, D) or 20 DAI (Figures 4D, F, Additional file 1: Figure S5A). To test whether reactive oxygen species (ROS) are involved in the cell death phenotype of TRV:NAG plants, leaf protoplasts prepared from VIGS lines (20 DAI) were incubated with H_2_DCFDA that becomes activated in the presence of H_2_O_2_ to produce green fluorescence. Accumulation of fluorescent H_2_DCFDA in TRV:NAG protoplasts was ~4.2-fold higher than in TRV control, indicating H_2_O_2_ accumulation (Figures 4G, H, Additional file 1: Figure S5A).

### Ultrastructural analyses of the cell death phenotype

Light (Additional file 1: Figure S3A, B) and transmission electron microscopy (TEM) (Additional file 1: Figure S3C-L) of transverse leaf sections revealed degenerating spongy mesophyll cells at early and late stages in TRV:NAG lines, in contrast to TRV control cells at 25 DAI. TEM also showed disintegrating chloroplasts (Additional file 1: Figure S3E, F, J) and mitochondria (Additional file 1: Figure S3L), ruptured vacuoles (Additional file 1: Figure S3E, F), and abnormal nuclei (Additional file 1: Figure S3H) in TRV:NAG lines, compared with TRV control (Additional file 1: Figure S3C, G, I, K). These results suggest that the *NbNAG*-induced cell death involves vacuole collapse leading to enzymatic degradation of organelles and cell contents, as demonstrated in PCD induced by other stimuli [23].

### NbNAG deficiency inhibits intracellular protein transport

**Figure 5:**
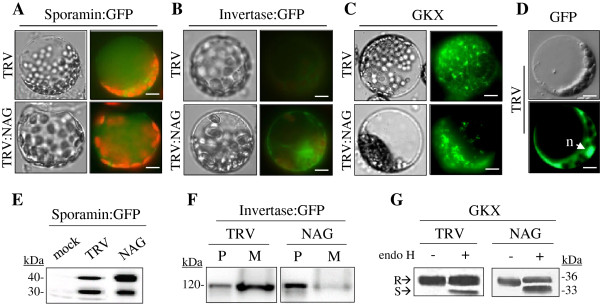
**Inhibition of intracellular trafficking of diverse cargo proteins. A** Vacuolar localization of sporamin:GFP, a fusion protein between sporamin and green fluorescent protein (GFP). Protoplasts isolated from TRV and TRV:NAG leaves were transformed with the sporamin:GFP construct. Fluorescent and bright field images are shown. Red fluorescence indicates chlorophyll autofluorescence. Scale bars: 10 μm. **B** Localization of invertase:GFP. Because invertase:GFP is a secretory protein, its fluorescence was not readily detected within protoplasts of TRV control . Scale bars: 10 μm. **C** Localization of GKX. GKX is a chimeric ER membrane marker [27]. Scale bars: 10 μm. **D** Localization of GFP control in the cytosol and the nucleus of TRV protoplasts. Scale bars:10 μm. **E** Trafficking assay of sporamin:GFP. Protein extracts prepared from protoplasts transformed with the sporamin:GFP construct were analyzed by western blotting using anti-GFP antibody. Mock indicates untransformed TRV protoplasts. **F** Trafficking assay of invertase:GFP. Protein extracts prepared from protoplasts (P) and medium (M) were analyzed by western blotting using anti-GFP antibody. **G** Endo H resistance of GKX glycans. Protein extracts from protoplasts transformed with the GKX construct were treated with endo H and analyzed by western blotting using anti-GFP antibody. *S* and *R* indicate endo H-sensitive GKX proteins (ER form) and endo H-resistant proteins (Golgi form), respectively.

Yeast 82-kDa Sec39p (Dsl3p) is localized to the ER [24], and associated with the syntaxin 18 complex that is involved in Golgi-to-ER retrograde transport [19]. Human NAG containing a Sec39p-homologous region appears to be the ortholog of yeast Sec39p despite the marked difference in their molecular sizes, and plays a similar role in the Golgi-ER retrograde trafficking [18]. NbNAG also contains the Sec39 domain (amino acid residues 595–1126), indicating that NbNAG may be involved in protein trafficking in a plant cell. To test this possibility, protoplasts isolated from TRV and TRV:NAG leaves (10 DAI) were transformed with *sporamin:GFP* (*Spo:GFP*) construct encoding a fusion protein between green fluorescent protein (GFP) and the N-terminal region of sporamin as a vacuolar reporter gene (Figure 5A). Previous experiments with *Arabidopsis* protoplasts showed that Spo:GFP is targeted to the central vacuole, where it is processed from a 40-kDa precursor to a 30-kDa form by proteolysis [25]. Consistent with the results, fluorescence microscopy revealed Spo:GFP in the central vacuole in both TRV and TRV:NAG protoplasts (Figure 5A), while GFP alone was localized in the cytosol and the nucleus in TRV protoplasts (Figure 5D). To measure trafficking efficiency, relative amounts of the precursor and the processed form of Spo:GFP were compared in transformed TRV and TRV:NAG protoplasts by immunoblotting using anti-GFP antibody (Figures 5E, Additional file 1: Figure S5B). In TRV protoplasts, ~44% of Spo:GFP was in the 40 kDa precursor form; however, in TRV:NAG protoplasts, the precursor form is visibly dominant, indicating that NbNAG deficiency leads to reduced vacuole trafficking of Spo:GFP.

We next examined the effects of NbNAG depletion on the secretory pathway using invertase:GFP, a chimeric protein consisting of full-length secretory invertase and GFP [26], as a reporter (Figure 5B). After transformation with the invertase:GFP construct, green fluorescent signals were not detectable in TRV protoplasts, indicating that invertase:GFP was secreted into the medium as previously observed [26]. However, invertase:GFP signal was readily detected in TRV:NAG protoplasts (Figure 5B). To confirm this finding, proteins were extracted from the protoplasts and the incubation medium, and analyzed by western blot analysis with anti-GFP antibody (Figures 5F, Additional file 1: Figure S5B). In TRV controls, invertase:GFP (~120 kDa) was mainly detected in the medium, while a minor portion of the protein remained in the protoplasts. In contrast, in the TRV:NAG line the fusion protein was predominantly present in the protoplasts, suggesting that NbNAG depletion blocked the secretion of invertase:GFP into the medium.

Next, we examined ER-Golgi transport using an artificial ER marker protein, GKX [27]. TRV protoplasts transformed with *GKX* exhibited a reticular network pattern of green fluorescence (Figure 5C) as observed previously [27]. However, TRV:NAG protoplasts expressing GKX displayed a punctate and aggregated fluorescence signal, suggesting disruption of the ER network. Since GKX contains an N-glycosylation site, we examined the sensitivity of the N-glycans of GKX to endoglycosidase H (endo H) treatment. It has been reported that the N-glycans of ER proteins are sensitive to endo H, while the N-glycans of Golgi proteins are resistant due to modification [28,29]. Protein extracts from TRV and TRV:NAG protoplasts were digested by endo H prior to western blotting with anti-GFP antibody (Figure 5G). In the TRV control, endo H digestion of GKX resulted in two bands, ~33 kDa endo H-sensitive GKX proteins (ER form) and ~36 kDa endo H-resistant proteins (Golgi form). These results indicate that a large part of GKX proteins was transported from ER to Golgi complex through anterograde trafficking. TRV:NAG lines also contained both the ER and the Golgi forms, but the ratio of the ER form to the Golgi form was higher than in TRV controls (Figures 5G, Additional file 1: Figure S5B). These results provide evidence that protein transport from ER to Golgi complex was inhibited by NbNAG deficiency.

### *NbNAG*-silencing causes ER stress

**Figure 6:**
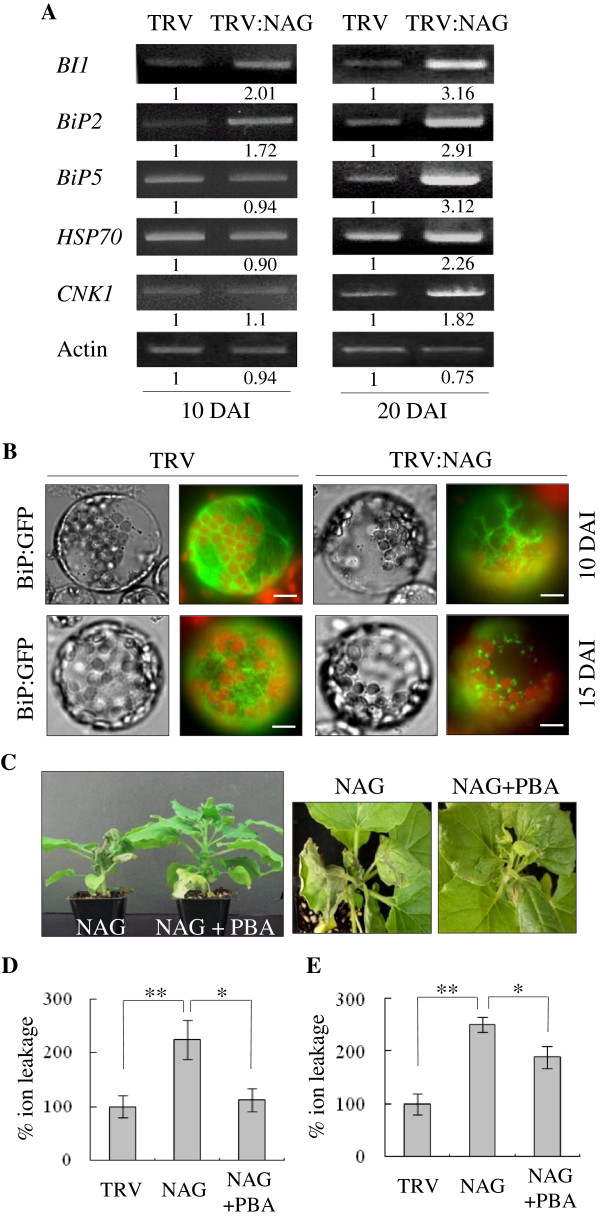
**ER stress phenotypes. A** Semiquantitative RT-PCR analysis of transcript levels of ER stress-related genes in TRV and TRV:NAG leaves at 10 and 20 DAI. Relative band intensity was shown below the bands. **B** Altered ER morphology. As an ER marker, BiP:GFP construct was transformed into leaf protoplasts of TRV and TRV:NAG lines at 10 and 15 DAI. BiP is a HSP70 chaperone located in the ER lumen. Red fluorescence indicates chlorophyll autofluorescence. Scale bars: 10 μm. **C** Effect of the chemical chaperone 4-phenyl butyric acid (PBA) on growth retardation and cell death phenotypes in TRV:NAG VIGS lines at 25 DAI. Representative plants are shown. **D** and **E** Relative ion leakage (%) of the 4th leaf above the infiltrated leaf (**D**) and the leaf near the shoot apex (**E**). Each value represents the mean ± SD of three replicates per experiment.

The abnormal ER morphology shown by GKX expression raised a possibility that NbNAG-deficient plants may have been subjected to ER stress. Transcriptome analysis in response to tunicamycin-induced ER stress in *Arabidopsis* revealed transcriptional up-regulation of the genes encoding BiPs, BI1 (Bax-Inhibitor 1), CNK1, and HSP70 [6,10]; therefore, we examined transcript levels of these ER stress-related genes in TRV and TRV:NAG lines. Semiquantitative RT-PCR revealed increased transcript levels of *BI1* and *BiP2* in TRV:NAG lines compared with TRV control at 10 DAI, while *BI1*, *BiP2*, *BiP5*, *CNK1*, and *HSP70* genes were all induced at 20 DAI (Figure 6A).

To further observe ER morphology, we transiently expressed a chimeric lumenal protein, BiP:GFP, a fusion protein between BiP (lumenal binding protein) and GFP, in protoplasts from TRV and TRV:NAG lines (Figure 6B). At 10 DAI, the fluorescent signal of BiP:GFP was localized in a reticular membranous network throughout the cytoplasm of both TRV control and TRV:NAG lines. At 15 DAI, the signal was punctate and fragmented in TRV:NAG lines indicating a disrupted ER network, while TRV exhibited normal ER morphology (Figure 6B).

We next tested whether the chemical chaperone 4-phenyl butyric acid (PBA) alleviates *NbNAG*-induced cell death (Figure 6C-E). PBA relieves ER stress by directly reducing the load of misfolded proteins retained in the ER and has been shown to mitigate ER stress-induced cell death in mammals and plants [6,30,31]. Treatment with PBA (1 mM) partially rescued the growth retardation and cell death phenotypes of TRV:NAG lines (Figure 6C). Elevated ion leakage, an indicator of cellular membrane leakage, in TRV:NAG lines was also alleviated by PBA treatment in the 4th leaf above the infiltrated leaf (Figures 6D, Additional file 1: Figure S5A) and in the leaf near the shoot apex (Figures 6E, Additional file 1: Figure S5A). These results suggest that the defects in protein transport may cause ER stress in NbNAG-deficient cells.

### Nuclear translocation of bZIP28

**Figure 7:**
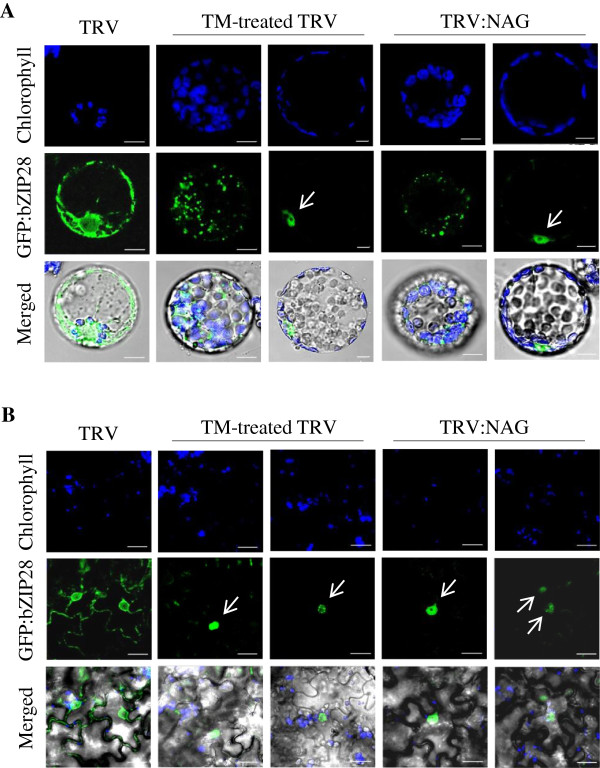
**Subcellular localization of GFP:bZIP28. A** TRV control, tunicamycin (TM)-treated TRV, and TRV:NAG plants (15 DAI) were infiltrated with *Agrobacterium* containing GFP:bZIP28 construct. After 24 h, protoplasts were isolated and localization of GFP fluorescent signals was examined by confocal laser scanning microscopy. Representative images of the protoplasts are shown. Scale bars: 10 μm. **B** After agroinfiltration of the GFP:bZIP28 construct, leaf epidermal cells were observed by confocal microscopy. Scale bars: 20 μm.

In *Arabidopsis*, tunicamycin-induced ER stress causes proteolytic processing and nuclear relocation of an ER membrane-associated transcription factor, bZIP28 [14,15]. We therefore tested whether silencing of *NbNAG* similarly induced nuclear relocation of bZIP28 from the ER membrane (Figure 7). A *GFP:bZIP28* construct in which *GFP* was fused to *Arabidopsis bZIP28* was transiently expressed in TRV and TRV:NAG leaves (15 DAI) by agro-infiltration. Protoplasts prepared from the infiltrated leaves were examined by confocal laser scanning microscopy (Figure 7A) and fluorescence microscopy (Additional file 1: Figure S4) to observe GFP:bZIP28 fluorescence in the mesophyll cells. In addition, infiltrated leaves were directly examined to observe the fluorescence in the epidermal cells (Figure 7B). Protoplasts and leaf epidermal cells from TRV control exhibited a network pattern of green fluorescence in the cytosol and in the nuclear periphery as previously observed [14]. However, protoplasts from TRV:NAG lines and tunicamycin (TM)-treated TRV lines (the positive control) frequently showed a punctuate and aggregated GFP signal in the cytosol, revealing an abnormal ER network (Figures 7A, Additional file 1: Figure S4). Furthermore, some of the protoplasts and the epidermal cells exhibited strong GFP fluorescence in the nucleus with little fluorescence remaining in the cytoplasm (Figures 7A, B, Additional file 1: Figure S4). Since nuclear relocation of GFP:bZIP28 was not observed in TRV protoplasts or TRV epidermal cells, these results suggest that NbNAG deficiency activates the ER stress response.

## Discussion

*Neuroblastoma-Amplified Gene* (*NAG*) encodes a large protein (~2,400 amino acids) containing the Sec39 domain in plants and mammals. In human neuroblastoma, *NAG* was found to be co-amplified with N-*myc* oncogene, of which genomic amplification correlates with aggressive tumor growth [16]. Despite the marked size difference, the NAG ortholog of yeast appears to be Sec39p (Dsl3p), a cytosolic protein of ~82 kDa, that is peripherally associated with the ER membrane [19]. Sec39p was first identified as an essential protein involved in ER-Golgi transport in a large-scale promoter shutoff analysis of essential yeast genes [32]. Further characterization revealed that Sec39p is a component of the Dsl1p complex that also includes Dsl1p, Tip20p, and ER-localized Q-SNARE proteins [19,33]. The Dsl1p complex is essential for retrograde traffic from the Golgi to ER, and consistently, *Sec39* showed strong genetic interaction with other factors required for Golgi-ER retrograde transport [19,34]. Interestingly, the temperature-sensitive *sec39* mutants also exhibited defects in forward transport between the ER and Golgi [19], consistent with the results by Mnaimneh et al. [32]. The phenotype can be explained by the fact that retrograde traffic plays an important role in recycling ER-resident proteins that have escaped from the ER [35,36]. Thus, a blocked retrograde pathway can result in failure to retrieve these proteins and lead to a concomitant block in anterograde transport.

Mammals possess functional orthologs of the components of the yeast Dsl1p complex, despite a low degree of sequence conservation between the yeast and mammalian counterparts [37]. Recently, it has been revealed that mammalian NAG is a subunit of the Syntaxin 18 complex involved in Golgi-to-ER retrograde trafficking and serves as a link between p31 and ZW10-RINT-1 in the mammalian ER fusion machinery [18]. Interestingly, silencing of *NAG* in human cell lines using RNA interference did not substantially disrupt Golgi morphology or the ER-to-Golgi anterograde trafficking, but caused defects in protein glycosylation [18]. In this study, we characterized *in vivo* effects of NAG deficiency in plants. *NAG* from *N. benthamiana*, *Arabidopsis*, and rice all encodes a ~2,400 kDa protein with the Sec39 domain that is mainly localized in ER, similar to the mammalian NAG. Knockdown of *NbNAG* using VIGS led to reduced protein transport between ER and Golgi, and a concomitant decrease in trafficking of the marker proteins to vacuole and to plasma membrane for secretion. Furthermore, NAG depletion *in planta* caused ER stress and PCD.

Our data suggest that ER stress caused by disrupted protein transport contributes to PCD activation in *NbNAG*-silenced plants. First, expression of the UPR-related genes such as *BiP2*, *BiP5, BI1* (Bax inhibitor-1), *HSP70*, and *CNK1* was up-regulated in *NbNAG*-silenced VIGS plants. *BiPs* encode ER-localized chaperones and have been used as marker genes for UPR activation in plants [12,38], and similarly, expression of *BI1*, *HSP70*, and *CNK1* was induced during tunicamycin-induced ER stress in *Arabidopsis*[6]. Second, NbNAG deficiency caused proteolytic processing and nuclear relocation of bZIP28 transcription factor, which is normally associated with the ER membrane. It has been shown that bZIP28 processing is activated by ER stress-inducing agents such as tunicamycin and dithiothreitol, but not by salt stress [14]. Third, the chemical chaperone PBA alleviated the growth retardation and cell death phenotypes of *NbNAG* VIGS plants. PBA suppresses ER stress and ER-mediated apoptosis by chemically enhancing the capacity of the ER to remove misfolded proteins in mammals and plants [6,30,31]. In addition, expression analyses of the *Arabidopsis NAG* promoter-*GUS* fusion gene showed that *AtNAG* promoter activity was stimulated in response to tunicamycin. Thus NbNAG appears to play a crucial role in protein transport during plant growth and development, and its deficiency causes ER stress response and subsequent activation of PCD. NbNAG-mediated cell death was first observed in young tissues containing actively dividing cells such as the shoot apex and vascular cambium before expanding to other tissues (Figure 3), consistent with the fact that young tissues have elevated demands for protein synthesis and transport.

Interestingly, mutations that inhibit the ER to Golgi trafficking have not always caused ER stress and PCD as observed in *NbNAG* VIGS plants. For example, a missense mutation in the COPII coat protein Sec24A caused the formation of aberrant tubular clusters of ER and Golgi membrane in *Arabidopsis* without inducing PCD [39]. In yeast and mammals, the COPII coat forms transport vesicles on the ER surface for the ER-Golgi anterograde trafficking, and the COPII coat protein Sec24A is believed to have a specific role in cargo selection via site-specific recognition of cargo signals [39]. In another case, overexpression of AtPRA1.B6, a prenylated Rab acceptor 1, resulted in inhibition of COPII vesicle-mediated anterograde trafficking but did not induce either ER stress or PCD [40]. The lack of PCD activation in the Sec24A mutant may be linked to the fact that the missense mutation caused only a partial loss of Sec24A function, affecting the anterograde trafficking of only a subset of cargos [39]. Total loss of Sec24A function instead led to an embryonic lethality, suggesting that the gene function is essential in *Arabidopsis*[39]. Similarly, because AtPRA1.B6 functions as a negative regulator of the anterograde transport of only a subset of proteins at the ER, the effect of its overexpression appeared to be rather specific and limited [40]. In this study, the severe consequences of NbNAG depletion suggest that NbNAG function in the protein transport pathway may be essential and/or common so that its deficiency severely disturbs the system. Alternatively, NbNAG may have additional, yet unidentified, functions, of which disruption induces PCD in a plant cell.

Although the underlying mechanism remains unclear, recent evidence indicates that mitochondria-dependent and –independent cell death pathways both play a role in ER stress-induced apoptosis [5,8,41]. Well-known regulators of mammalian apoptosis, such as the Bcl-2 family and caspases, are activated during ER stress [5,41]. Bcl-2, Bax and Bak reside in the ER membrane as well as in the mitochondrial outer membrane, regulating homeostasis and apoptosis in the ER [5]. Overexpression of Bcl-2 or deficiency of the proapoptotic proteins Bax and Bak confers protection against ER stress-induced apoptosis, indicating that Bcl-2 family members participate in the integration of apoptotic signals between the ER and mitochondria [42,43]. In this study, NbNAG-induced cell death showed apoptotic hallmarks, such as nuclear DNA fragmentation, decreased mitochondrial membrane potential, and excessive production of reactive oxygen species. These apoptotic features are similar to the phenotypes of ER stress-induced PCD in *Arabidopsis* roots and soybean cell cultures caused by tunicamycin and cyclopiazonic acid, respectively [6,44]. In particular, the decreased mitochondrial membrane potential strongly indicates involvement of mitochondria in NbNAG-induced PCD, at least at the stage we examined. It will be important to determine whether several pro-apoptotic pathways simultaneously function to commit the cell to death during ER stress, and how the different signals from the ER are integrated to activate PCD in plants.

## Conclusion

*Nicotiana benthamania Neuroblastoma-Amplified Gene* (*NbNAG*) encodes an ER-localized protein of 2,409 amino acids that contains the secretory pathway Sec39 domain. NbNAG plays a role in protein transport pathway, and NbNAG deficiency resulted in ER stress and programmed cell death, presumably caused by a blocked secretion pathway. These results suggest that NAG, a conserved protein from yeast to mammals, plays an essential role in plant growth and development.

## Methods

### Virus-induced gene silencing

Virus-induced gene silencing was performed as described [22,45,46]. *NbNAG* cDNA fragments were PCR-amplified and cloned into the pTV00 vector containing part of the tobacco rattle virus (TRV) genome using the following NbNAG specific primers: NbNAG N1 (5'-aagcttatggaggaatcaact-3' and 5'-gggcccttggatcttgattga-3') and NbNAG N2 (5'-aagcttgttacagaatggaat-3' and 5'-gggcccagatatgccaagtcc-3'). The recombinant pTV00 plasmids and the pBINTRA6 vector containing RNA1 required for virus replication were separately transformed into *Agrobacterium tumefaciens* GV3101 strain. After grown to saturation, the *Agrobacterium* culture was centrifuged and resuspended in 10 mM MgCl2, 10 mM MES and 150 μM acetosyringone, and kept at room temperature for 2 h. Separate cultures containing pTV00 and pBINTRA6 were mixed in a 1:1 ratio. The third leaf of *N. benthamiana* (3-week old) was pressure-infiltrated with the mixed *Agrobacterium* suspension as described [47]. Since the *Agrobacterium* infiltration into *N. benthamiana* leaves causes systemic spread of gene silencing signal into upper leaves and vasculature of the growing plants, the gene silencing phenotypes were observed in the newly emerged tissues at approximately 2 weeks after infiltration. The 4th leaf above the infiltrated leaf was used for semiquantitative RT-PCR.

### Cloning of *NbNAG*

The partial *NbNAG* cDNA used in the VIGS screening was ~1.8 kb in length and corresponded to the N-terminal end of the predicted protein. We searched *N. benthamiana* and *N. tabacum* EST databases using *Arabidopsis* and rice *NAG* sequences to find a *N. tabacum* clone (BP130717) containing the C-terminal end of the *NAG* gene. Using a long range PCR amplification kit (Qiagen), we amplified a ~7.4 kb cDNA fragment using cDNA synthesized from *N. benthamiana* seedling RNA as template and primers corresponding to the 5’-termimal sequence of the ~1.8 kb cDNA (5'-caccctcaaggagagatggagaaagcag-3') and the 3’-terminal sequence of the tobacco clone (5'-agcttctgctcgacagtatccaag-3'). The amplified fragment was cloned into TOPO cloning vector (TOPO^R^ XL PCR cloning kit) and sequenced.

### GUS histochemical assay

The *AtNAG* promoter sequence was PCR-amplified from *Arabidopsis* genomic DNA using primers 5'-aagcttgtggaatattattttcaa-3' and 5'-ggatccgatcaatcgagatcgatc-3'. The 1,100 bp promoter was cloned into pBI101 vector using HindIII/BamHI sites to generate the *AtNAG* promoter-*GUS* fusion gene. The recombinant Ti-plasmid was introduced into *A. tumefaciens* LBA4404 for *Arabidopsis* transformation. GUS staining of the transgenic *Arabidopsis* lines was performed as described [48].

### RNA isolation and semiquantitative RT-PCR analysis

Semiquantitative RT-PCR was performed with RNA isolated from the fourth leaf above the infiltrated leaf as described [45], with 15–35 cycles of amplification. The endogenous *NbNAG* transcript was detected using the primers 5'-ctgggagttcacctcctcca-3' and 5'-gcgagctcaacccaagaagt-3'. To detect *BiP* and *HSP70* transcripts, the following primers were designed based on the published *N. benthamiana* and tobacco cDNA sequences: *BI1* (5'-gcaatcgctggagttacgat-3' and 5'-ccaaggtgtgc cttctcaat-3'), *BiP2* (X60059; 5'-agtgcaacagctcctgaagga-3' and ctgttacgggcatcaatcctc-3'), *BiP5* (X60058; 5'-tggaagagacgcgcatccttg-3' and 5'-gaccaggatgttcttttcacc-3'), *HSP70* (NP1072062; 5'-cactctcatccactgctcaga-3' and 5'-gtggtcttgtcctcagcagag-3'), and actin (5'-tggactctggtgatggtgtc-3' and 5'-cctccaatccaaacactgta-3').

### Histochemical analyses

Tissue sectioning, light microscopy, and transmission electron microscopy were carried out using the fourth or fifth leaf above the infiltrated leaf of the VIGS lines as described [22].

### Measurement of *in vivo* H_2_O_2_, mitochondrial membrane potential, and DNA fragmentation analysis

These experiments were carried out as described [22].

### Immunolabeling

Anti-NbNAG antibodies were generated in rabbits against an N-terminal region (351 amino acids) of NbNAG using antibody production services of AbFrontier (http://www.abfrontier.com). Preparation of BY-2 cells and immunofluorescence were performed as described [49,50]. Fixed and permeabilized BY-2 cells were immunolabeled with 1:500 dilution of anti-NbNAG antibodies. Then the cells were incubated with 1:1000 dilution of Alexa Fluor® 594-conjugated anti-rabbit IgG antibodies (Molecular Probes). To mark the ER, the cells were briefly stained with 1 μM ER Tracker™ Blue-White DPX (Molecular Probes). Then the BY-2 cells were observed under a confocal laser scanning microscope (Carl Zeiss LSM 510) with optical filters LP 560 (excitation 543 nm, emission 560–615 nm) and BP420-480 (excitation 405 nm, emission 461 nm) for Alexa Fluor 594 and ER Tracker, respectively. The BY-2 cells were also observed under a fluorescence microscope (Olympus IX71).

### Transient expression of reporter proteins

Cloning of Sporamin:GFP, Invertase:GFP, GKX, and BiP:GFP was previously described [25,26,40,51]. Plasmids (15 μg) were introduced into protoplasts prepared from the fourth leaf above the infiltrated leaf of VIGS lines at 10 days after infiltration (DAI) by polyethylene glycol mediated transformation. Expression of the fusion constructs was monitored at various time points after transformation, and images were captured with a cooled CCD camera and a Zeiss Axioplan fluorescence microscope (Jena, Germany) according to [52].

### Protein preparation and western blot analysis

To prepare cell extracts from protoplasts, transformed protoplasts were subjected to repeated freeze and thaw cycles in lysis buffer (150 mM NaCl, 20 mM Tris-Cl, pH 7.5, 1 mM EDTA, 1 mM EGTA, 3 mM MgCl2, 0.1 mg/ml antipain, 2 mg/ml aprotinins, 0.1 mg/ml E-64, 0.1 mg/ml leupeptin, 10 mg/ml pepstatin, and 1 mM phenylmethylsulfonyl fluoride) and then centrifuged at 7,000 *g* at 4°C for 5 min [51]. To extract proteins from culture medium, cold TCA (100 μl) was added to the medium (1 ml), and protein aggregates were precipitated by centrifugation at 10,000 *g* at 4°C for 5 min. The protein aggregates were dissolved in the same volume of lysis buffer used to prepare total protoplast proteins. Western blot analysis was performed using anti-GFP antibody (Clontech, Palo Alto, CA) as described previously [51].

### Endo H treatment

Endo H treatment was performed according to [52]. Protein extracts were prepared from transformed protoplasts and denatured in denaturation solution (1% SDS, 2% β-mercaptoethanol) by 10 min incubation at 100°C. Denatured proteins were incubated with 2 mg/ml endo H (Roche Diagnostics) in G5 buffer (50 mM sodium citrate, pH 5.5) at 37°C for 2 h. Samples were subjected to SDS-PAGE and analyzed by western blotting with anti-GFP antibody (Clontech, Palo Alto, CA).

### Agrobacterium-mediated transient expression

Agro-infiltration was carried out as described [53]. Protoplasts were prepared from the infiltrated leaves of TRV and TRV:NAG plants (15 DAI) 24 h post-infiltration, and GFP:bZIP28 fluorescence was observed by fluorescence microscopy.

### Sodium 4-phenylbutyrate (PBA) treatment and ion leakage measurement

Each experiment was performed three times using 10 plants per treatment. Starting at 5 DAI, TRV:NAG plants were irrigated every other day with 1 mM PBA or distilled water until 25 DAI. Leaf discs were prepared from multiple independent TRV:NAG plants for analysis. Sample preparation and conductivity measurements were carried out as described [45].

### Measurement of band intensity

The band intensity in the RT-PCR and immunoblotting analyses was measured using the AnalySIS LS Research program (Olympus).

### Statistical analyses

Two-tailed Student’s *t*-tests were performed using the Minitab 16 program (Minitab Inc.; http://www.minitab.com/en-KR/default.aspx) to investigate the statistical differences between the responses of the samples. Significant differences between control and other samples were indicated by one (*P* ≤ 0.05) or two (*P* ≤0.01) asterisks.

### Accession number

Genbank accession number: EU602317 (NbNAG)

## Competing interest

The authors declare that they have no competing interests.

## Authors’ contributions

J-YL and SS performed most of the experiments and analyzed the results. HSK performed the promoter-GUS assays. HK and IH performed the protein trafficking assays. YJK performed the histological analyses. IH and YJK discussed the results and commented on the manuscript. H-SP designed the experiments and wrote the manuscript. All authors read and approved the final manuscript.

## Supplementary Material

Additional file 1: Figure S1 NAG sequence alignment. **Figure S2.** Nuclear morphology and mitochondrial membrane integrity at 10 DAI. **Figure S3.** Ultrastructural analyses using transmission electron microscopy (TEM). **Figure S4.** Fluorescence microscope images of GFP:bZIP28 localization. **Figure S5.** Measurement of fluorescence intensity and band intensity. Click here for file
