# Supplementary material for: Silencing of Nicotiana benthamiana Neuroblastoma-Amplified Gene causes ER stress and cell death
Source: BMC Plant Biol. 2013 Apr 27;13:69. doi: 10.1186/1471-2229-13-69 (PMC3654999; doi:10.1186/1471-2229-13-69)
Supplement: Additional file 1: Figure S1 — NAG sequence alignment. Figure S2. Nuclear morphology and mitochondrial membrane integrity at 10 DAI. Figure S3. Ultrastructural analyses using transmission electron microscopy (TEM). Figure S4. Fluorescence microscope images of GFP:bZIP28 localization. Figure S5. Measurement of fluorescence intensity and band intensity. [file 1471-2229-13-69-S1.pdf]

Figure S1

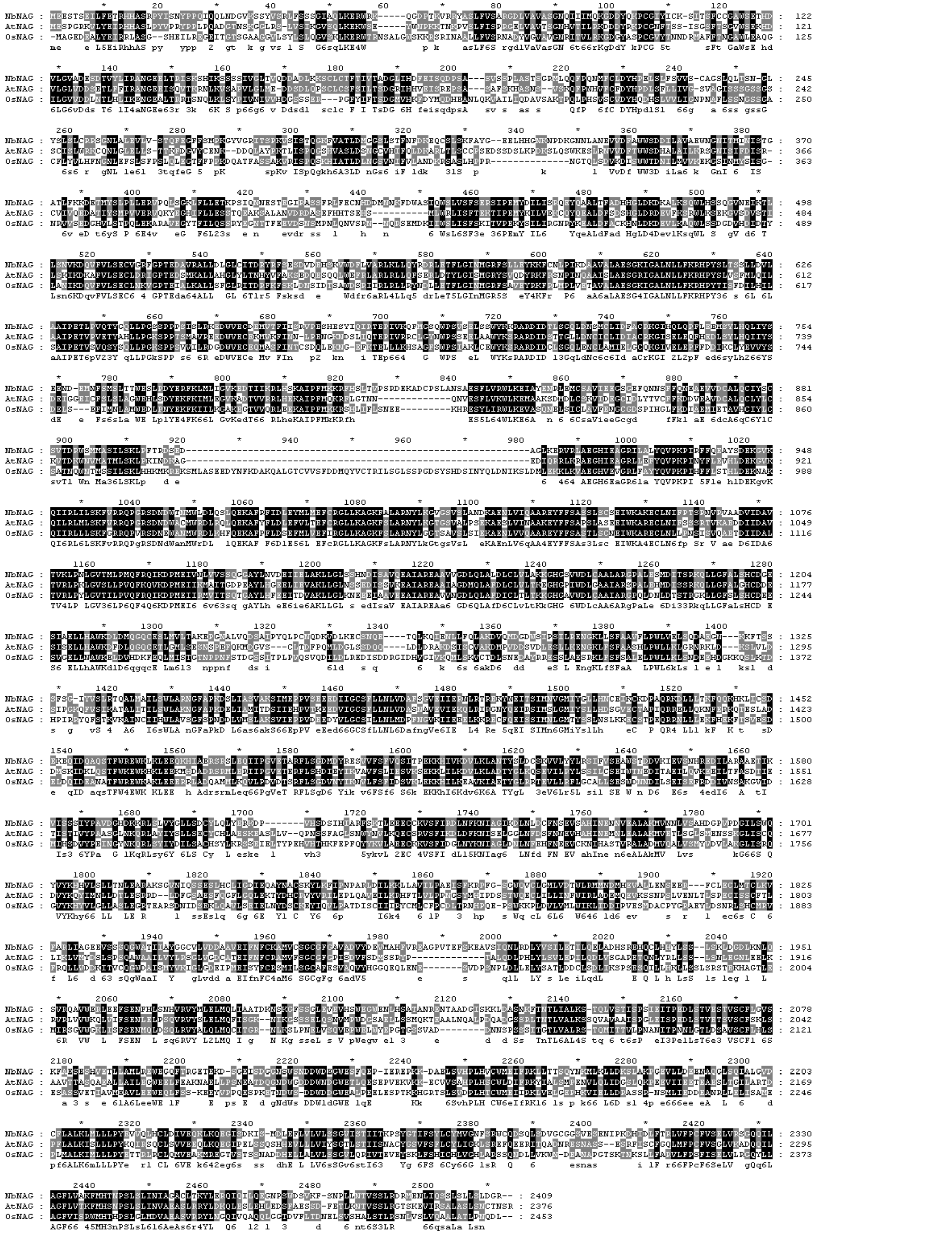

Figure S1 NAG sequence alignment.

Deduced amino acid sequences of *NbNAG* are aligned with related sequences from *Arabidopsis* (AtNAG: At5g24350), and rice (OsNAG: NM_001072983). The numbers indicate amino acid residues. Residues that are conserved between the sequences are boxed in black or gray based on the degree of conservation.

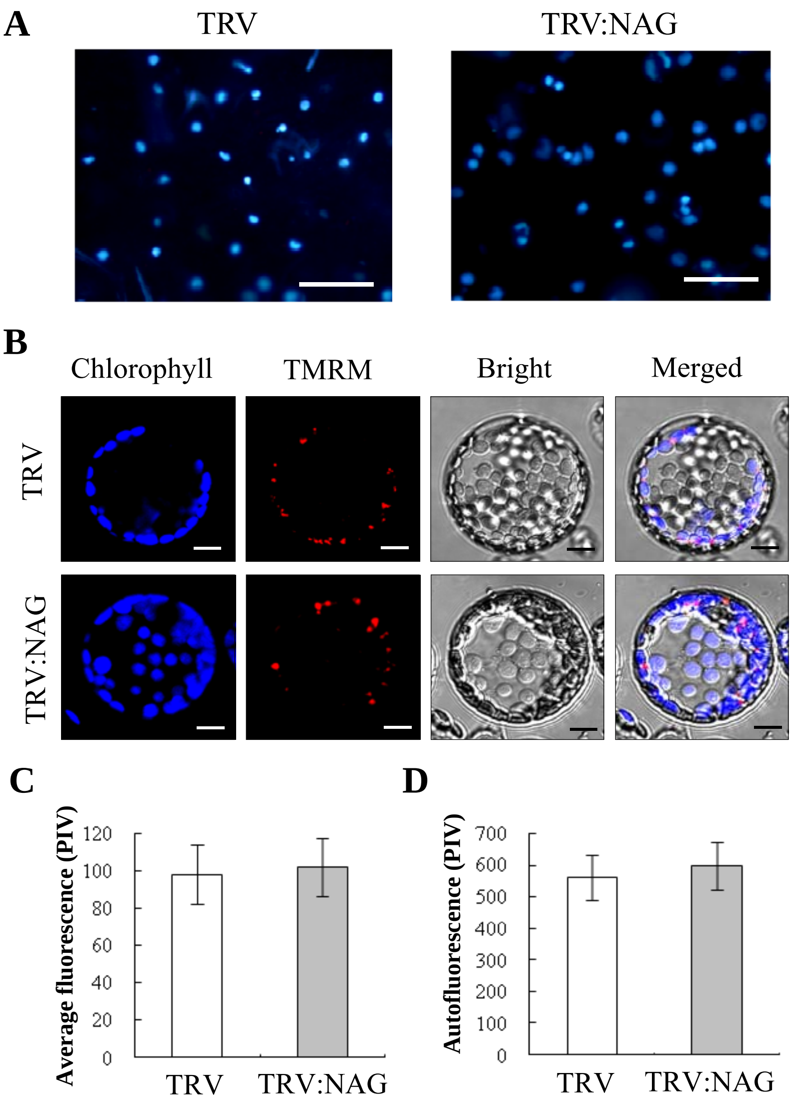

Figure S2 Nuclear morphology and mitochondrial membrane integrity at 10 DAI.

A Fluorescence microscopy of abaxial leaf epidermal cells from TRV control and TRV:NAG lines (10 DAI) after DAPI staining. Scale bars: 100 μm.

B Leaf protoplasts from VIGS lines (10 DAI) were observed after staining with TMRM (200 nM). Scale bars: 10 μm.

C TMRM fluorescence was quantified by pixel intensity. Data points represent means ± SD of 20 individual protoplasts. PIV, pixel intensity values.

D Chlorophyll autofluorescence was quantified by pixel intensity. Data points represent means ± SD of 20 individual protoplasts.

Figure S3

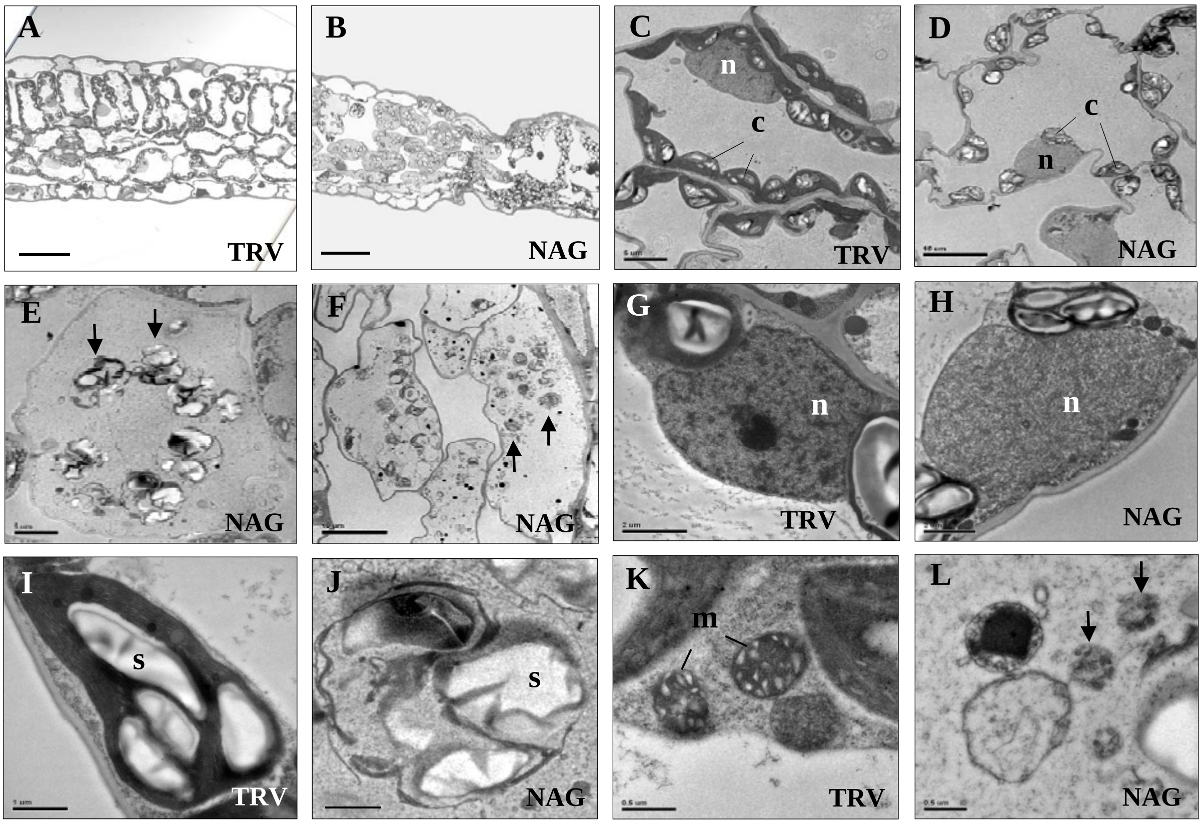

Figure S3 Ultrastructural analyses using transmission electron microscopy (TEM).
Light (**A**, **B**) and transmission electron micrographs (**C**-**L**) of leaf transverse sections from TRV and TRV:NAG lines at 25 DAI. Spongy mesophyll cells (**C**-**F**), nucleus (**G**, **H**), chloroplast (**I**, **J**), and mitochondria (**K**, **L**) are shown. Disintegrating chloroplasts and mitochondria are indicated by *arrows* in (**E**, **F**) and (**L**), respectively. Scale bars: 100 µm (**A**, **B**); 10 µm (**D**, **F**); 5 µm (**C**, **E**); 2 µm (**G**, **H**); 1 µm (**I**, **J**); 0.5 µm (**K**, **L**). *c*, chloroplast; *n*, nucleus; *s*, starch; *m*, mitochondria.

Figure S4

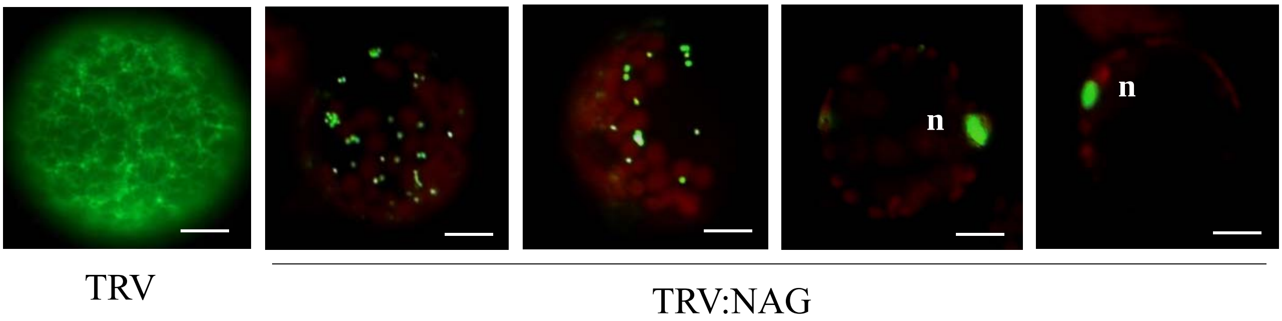

Figure S4 Fluorescence microscope images of GFP:bZIP28 localization.

Protoplasts were isolated from TRV and TRV:NAG plants infiltrated with *Agrobacterium* containing *GFP:bZIP28*, and localization of green fluorescent signals was examined by fluorescence microscopy. Representative images of the protoplasts are shown. It has been reported that ER stress causes proteolytic processing and nuclear relocation of the ERmembrane-associated bZIP28 transcription factor [14, 15]. *n*, nucleus. Scale bars: 10 μm.

A Values

	TRV	TRV:NAG	TRV:NAG + PBA
TMRM (PIV)	80±12.1	19.3±5.1	
Autofluorescence (PIV)	560±69.6	598±74.8	
H_2DCFDA (PIV)	15±6.6	54±13.6	
% ion leakage (4th leaf)	100±20.2	224±34.2	112±21.5
% ion leakage (shoot apex)	100±19.7	250±14.1	189±21.0

B Band intensity in immunoblots (%)

		TRV	TRV:NAG
Sporamin:GFP	precursor	44.04	60.88
	mature	55.96	39.12
Invertase:GFP	protoplast	29.23	74.78
	medium	70.77	25.22
GKX (+ endoH)	ER form	22.38	51.91
	Golgi form	77.62	48.09

Figure S5 Measurement of fluorescence intensity and band intensity

A Actual values of TMRM staining (Figure 4E), autofluorescence (Figure 4F), H_2DCFDA staining (Figure 4H), and % ion leakage (Figure 6D, E) are presented.

B Band intensity in the immunoblots for Sporamin:GFP transport (Figure 5E), Invertase:GFP transport (Figure 5F), and GKX transport (Figure 5G) are presented.
